# Phase Changes of Monosulfoaluminate in NaCl Aqueous Solution

**DOI:** 10.3390/ma9050401

**Published:** 2016-05-21

**Authors:** Seyoon Yoon, Juyoung Ha, Sejung Rosie Chae, David A. Kilcoyne, Yubin Jun, Jae Eun Oh, Paulo J.M. Monteiro

**Affiliations:** 1 Department of Civil Engineering, Kyonggi University, Suwon 16227, Korea; yoonseyoon@kyonggi.ac.kr; 2 School of Urban and Environmental Engineering, Ulsan National Institute of Science and Technology (UNIST), 50 UNIST-gil, Ulsan 44919, Korea; ssjun97@gmail.com; 3 School of Environmental and Life Sciences, Kean University, Union, NJ 07083, USA; juyoung@gmail.com; 4 Department of Civil and Environmental Engineering, University of California, Berkeley, CA 94720, USA; busyrosy@berkeley.edu (S.R.C.); monteiro@berkeley.edu (P.J.M.M.); 5 Advanced Light Source, Lawrence Berkeley National Laboratory, Berkeley, CA 94720, USA; alkilcoyne@lbl.gov

**Keywords:** cement chemistry, STXM, XANES, chloride, Kuzel’s salt, Friedel’s salt, monosulfate

## Abstract

Monosulfoaluminate (Ca_4_Al_2_(SO_4_)(OH)_12_∙6H_2_O) plays an important role in anion binding in Portland cement by exchanging its original interlayer ions (SO_4_^2−^ and OH^−^) with chloride ions. In this study, scanning transmission X-ray microscope (STXM), X-ray absorption near edge structure (XANES) spectroscopy, and X-ray diffraction (XRD) were used to investigate the phase change of monosulfoaluminate due to its interaction with chloride ions. Pure monosulfoaluminate was synthesized and its powder samples were suspended in 0, 0.1, 1, 3, and 5 M NaCl solutions for seven days. At low chloride concentrations, a partial dissolution of monosulfoaluminate formed ettringite, while, with increasing chloride content, the dissolution process was suppressed. As the NaCl concentration increased, the dominant mechanism of the phase change became ion exchange, resulting in direct phase transformation from monosulfoaluminate to Kuzel’s salt or Friedel’s salt. The phase assemblages of the NaCl-reacted samples were explored using thermodynamic calculations and least-square linear combination (LC) fitting of measured XANES spectra. A comprehensive description of the phase change and its dominant mechanism are discussed.

## 1. Introduction

The NaCl aqueous solution is a frequent environment for cement-based materials. For example, marine and offshore concrete structures are generally exposed to seawater and thus corrosion of reinforcing steel bars by chloride attack is of significant concern in the deterioration of reinforced concrete (RC) structures [1,2]. It is also of great interest to study the interaction between hydrated phases of Portland cement and NaCl aqueous solution. Among the hydrated cement phases, Al_2_O_3_-Fe_2_O_3_-mono (AFm) phases, which have a layered double hydroxide (LDH) structure, are important “sinks” for chloride ions. The binding process of the AFm phases has been extensively studied [3,4,5,6,7,8,9,10,11,12,13].

The structure of AFm phases consist of positively charged main layers (Ca_2_Al(OH)_6_)^+^ and negatively charged interlayer components (*X*·nH_2_O)^1− or 2−^, where *X* can be either a monovalent or divalent anion, as well as neutral water molecules. In hydrated Portland cement, one of the most common AFm phases is a calcium monosulfoaluminate hydrate (or simply called, monosulfoaluminate) (Ca_4_Al_2_(SO_4_)(OH)_12_∙6H_2_O), which contains SO_4_^2−^ in the *X* position and the sulfate ions are provided from gypsum (CaSO_4_∙2H_2_O) or anhydrite (CaSO_4_), added to Portland cement to control setting properties.

Recent studies suggested that the reaction of chloride ions with monosulfoaluminate mainly resulted in Friedel’s salt (Ca_4_Al_2_Cl_2_(OH)_12_∙4H_2_O), which contains Cl^−^ in the *X* position [6,12,13,14,15,16,17], and dissolution/precipitation and ion exchange were the responsible mechanisms for the Friedel’s salt formation [16]. In the ion exchange, sulfate ions were released into pore solution from monosulfoaluminate due to the chloride binding:

(1) $$\left[R-SO_{4}\right]+2Cl^{-}↔\left[R-2Cl\right]+S{O_{4}}^{2-}$$

where *R* represents ion exchange sites. Beside Friedel’s salt, a chloro-sulfate AFm phase, known as Kuzel’s salt (Ca_4_Al_2_(SO_4_)_0.5_Cl(OH)_12_∙6H_2_O), was also observed when chloride ions were present [6,12,13] and a possible precipitation of Al_2_O_3_-Fe_2_O_3_-tri (AFt) phase (e.g., ettringite, Ca_6_Al_2_(SO_4_)_3_(OH)_12_∙26H_2_O) was suggested based on X-ray Diffraction (XRD) analysis at low concentrations of chloride [6]. In the exchange reaction, various conditions, such as cations, alkalinity, portlandite, mixed sulfate and chloride concentrations, influence on the rate and process of the chloride binding [18,19,20,21,22]. Balonis *et al.* [13] reported that there was no solid solution phase that gradually changed in chemical composition from monosulfoaluminate to Friedel’s salt except Kuzel’s salt, which is an ordered phase that consists of alternatively placed chloride ions in one layer and sulfate ions in another. However, Mesbah *et al.* [23] made a conflicting conclusion that a solid solution was present between those two phases after performing Rietveld XRD analyses. Therefore, in order to develop a proper adsorption model, we should decide whether to choose a discontinuous adsorption of phase transition, or a continuous adsorption isotherm. In particular, if the phase transition discontinuously occurs at a specific chemical environment, it is unlikely that the continuous adsorption isotherms (e.g., Freundlich or Langmuir isotherms) can properly describe the adsorption process of chloride ions by monosulfoaluminate. For this reason, a further study is necessary to understand the detailed process of the phase change due to the chloride binding for monosulfoaluminate in saline environments.

This study provides fundamental information to quantitatively estimate the chloride-binding capacity and isotherms of cement-based materials. Pure monosulfoaluminate was synthesized and reacted with different NaCl concentrations of aqueous solutions. X-ray diffraction (XRD) and synchrotron scanning transmission X-ray microscopy (STXM) with concurrent measurements of X-ray absorption near-edge structure (XANES) spectroscopy were used to quantitatively monitor the phase change and the morphology of the reacted monosulfoaluminate. In addition, thermodynamic calculations were conducted to theoretically deduce a possible phase change of monosulfoaluminate under a saline environment using a previously reported database for cementitious substances and compared with the quantified results obtained from the XRD and XANES analysis.

## 2. Experimental

### 2.1. Sample Preparation

Pure AFm phases (*i.e.*, monosulfoaluminate, Friedel’s salt, and Kuzel’s salt) were prepared according to the methods of Matschei *et al.* [24], and ettringite was synthesized using the method described by Perkins *et al.* [25]. All the synthesized pure phases were used as references for the XANES analyses, and monosulfoaluminate was also used as a raw material for the reaction with chloride ions.

To produce the AFm phases, tricalcium aluminate (C_3_A, Ca_3_Al_2_O_6_) was prepared as a precursor; C_3_A was synthesized from the stoichiometric sintering of CaCO_3_ with Al_2_O_3_ at a 3:1 molar ratio in a muffle furnace at 1450 °C with subsequent quenching.

Pure monosulfoaluminate was synthesized from the stoichiometric mixing of 1 mol C_3_A and 1 mol CaSO_4_ in deionized water; thereafter, the mixture was stirred for 7 days at 85 °C. Then, the sample was filtered under a nitrogen atmosphere and dried in a vacuum chamber.

Friedel’s salt was prepared by mixing C_3_A and CaCl·2H_2_O at a 1:1 molar ratio with deionized water and then stirring in a sealed container for one month at 23 ± 2 °C with subsequent filtration and drying.

Kuzel’s salt was made by adding C_3_A, CaCl_2_·2H_2_O, and CaSO_4_ in stoichiometric quantities to deionized water. The mixture was sealed to prevent carbonation and stirred for 6 months at 23 ± 2 °C before filtration and drying.

For ettringite synthesis, two reactant solutions were firstly made as 6.65 g Al_2_(SO_4_)_3_·18H_2_O in 100 mL deionized water and 4.44 g Ca(OH)_2_ in 250 mL deionized water. These reactant solutions were mixed together in a nitrogen environment and diluted to 500 mL with more water and 0.5 mL of 1 M NaOH solution. The mixture was sealed and stirred for 48 h at 60 °C before filtration and drying.

Lastly, the synthesized monosulfoaluminate was reacted with chloride ions by suspending 0.02 g of synthesized monosulfoaluminate in 1 mL of 0, 0.1, 1, 3, and 5 M NaCl solutions for 7 days at 23 ± 2 °C. Thereafter, the samples were filtered and dried in vacuum desiccators before experiments.

### 2.2. XRD, STXM, and XANES

The NaCl-reacted samples were characterized to identify constituting phases using powder XRD (Rotaflex RU200B, Rigaku, Tokyo, Japan) with CuKα radiation (λ = 1.5418 Å) at room temperature. The XRD patterns were analyzed with X’Pert HighScore Plus software package [26].

The STXM measurements reported herein were conducted at Beamline 5.3.2, which is a scanning transmission microscope that uses a monochromatic X-ray beam, at the Advanced Light Source (ALS) in Lawrence Berkeley National Laboratory. Beamline 5.3.2 simultaneously takes a STXM image at a given energy and record XANES spectra for a specific chemical element. In the XANES, X-ray energy is required to remove an electron from K-shell or L-shell of a given element. The particular X-ray energies correspond to the K-edge and the L-edge of the specific element, respectively. The least-square LC fitting of the collected XANES spectra can be used for phase quantification of mixture sample.

The pure AFm (synthesized in U.C. Berkeley, San Francisco, CA, USA; and the University of Aberdeen, Aberdeen, UK) phases (*i.e.*, monosulfoaluminate, Friedel’s salt, and Kuzel’s salt) and the NaCl-reacted monosulfoaluminate were mounted on 500 × 500 µm^2^ Si_3_N_4_ windows. To take STXM images, X-ray beam was focused on the mounted samples using a zone plate, and then two-dimensional images were collected by scanning the samples at a fixed photon energy. The ALS STXM Beamline allows data collection in modes of single energy imaging, line scanning, and multiple energy images (known as “stacks”) by scanning a sample in the *x*- and *y*-directions (single energy image or stacks) or *x*-direction (line scan). In the present study, stacks were obtained with energy increments (0.1 eV) over the energy range of Ca L_III,II_-edge in a stepwise manner. The XANES spectra were recorded from these image stacks. The procedure for collecting stack images consisted of subsequently taking STXM images at every energy level for the Ca L_III,II_-edge on a selected area, in which one pixel was as small as 25 to 30 nm. Normalization and background correction for the XANES spectra were performed by dividing each spectrum by a background spectrum taken at a sample-free area on the Si_3_N_4_ window. Axis 2000 software (version 2.1) [27] was used to align the stack images and extract XANES spectra from the stack images. Further details on the experimental method can be found in an earlier study [28].

### 2.3. Spectral Deconvolution for Quantification and Thermodynamic Modeling

The least-square LC fitting, implemented in the IFEFFIT package (ATHENA) [29], was performed to deconvolute the XANES spectra of the NaCl-reacted samples into the XANES spectra of reference samples (*i.e.*, synthesized monosulfoaluminate, Friedel’s salt, and Kuzel’s salt). The *R*-factor was used as an indicator of the quality of the fitting:

(2) $$R=\frac{\sum \text{​}{\left(\mu_{\text{i,exp}}-\mu_{\text{i,fit}}\right)}^{2}}{\sum \text{​}{\left(\mu_{\text{i,exp}}\right)}^{2}}$$

where µ_i,exp_ is the measured absorption of the sample and µ_i,fit_ is the fitted absorption.

Thermodynamic calculations were carried out under the assumption that no solid solution existed between monosulfoaluminate and Friedel’s salt except Kuzel’s salt using the geochemical code PHREEQC [30], and the thermodynamic database HATCHES version NEA 15 [31]. There are α and β-phases of Friedel’s salt, and the structural phase transition from α to β occurs at 34 °C [32,33,34,35]. In the present study, the α-phase was used because all experiments were performed at room temperature. The experiments and calculations in this study were maintained at room temperature. The solubility constants and densities of the AFm and AFt compounds were obtained from the thermodynamic data reported in earlier studies [24,36,37].

## 3. Results and Discussion

### 3.1. Reference XANES Spectra of Synthesized AFm and AFt Phases

AFm phases have a layered structure that mainly consists of octahedral Ca(OH)_6_ sheets; however, one-third of Ca atoms are replaced with Al, and Fe(III) is present as a relatively minor substituent. Calcium cations are present in a seven-fold coordination with a distorted octahedral Ca(OH)_6_ polyhedra, and additional water molecules exist in the interlayer space. These water molecules may interact with interlayer anions, such as SO_4_^2−^, OH^−^, Cl^−^, and CO_3_^2−^. Therefore, probing the nearest atomic neighbors and coordination environments around the Ca (or Al) atoms can be an effective way of identifying AFm phases, and the XANES spectra can provide such information to characterize AFm phases. However, Wieland *et al.* [38] observed no difference in Al K-edge XANES spectra between AFm phases despite the different chemical compositions and structures because the changes in coordination environments around Al atom in AFm compounds might be less sensitive to the variations in the interlayer substitutes, while Naftel *et al.* [39] reported that Ca L_III,II_-edge of XANES spectra was useful to detect differences in magnitude of crystal field of calcium compounds. Thus, because the synthesized AFm phases had different electro-negativities of interlayer anions, we collected the Ca L_III,II_-edge XANES spectra of the synthesized phases in this study.

**Figure 1:**
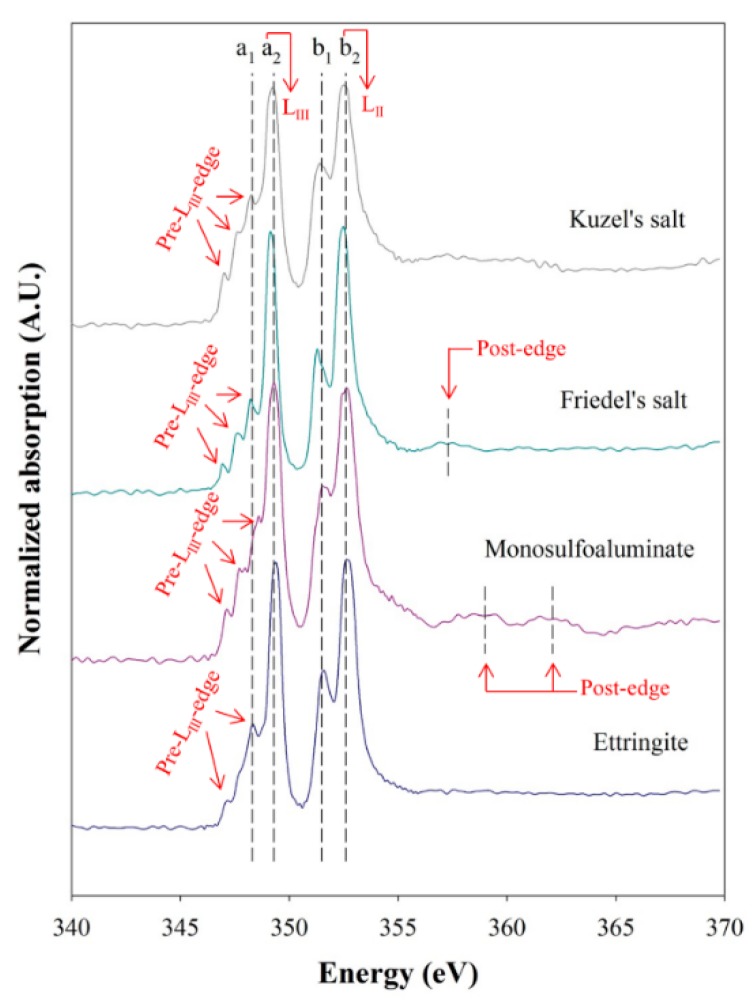
XANES (X-ray Absorption Near Edge Structure) reference spectra of Ca L_III,II_-edge of synthesized Kuzel’s salt, Friedel’s salt, monosulfoaluminate, and ettringite. The broken lines indicate spectral features.

Figure 1 shows the collected Ca L_III,II_-edge XANES spectra of the synthesized Kuzel’s salt, Friedel’s salt, monosulfoaluminate, and ettringite, which were used as reference spectra for deconvolution of XANES. These spectra were extracted and normalized using aXis 2000 and IFEFFIT software package [27,29,40]. We labeled the main peaks (*a*_1_, *a*_2_, *b*_1_, and *b*_2_). Detailed information is listed in Table 1. All the samples showed two well-resolved peaks corresponding to L_III_ at ~349 eV (=*a*_2_) and L_II_ at ~353 eV (=*b*_2_). The multiple peaks before the Ca L_III_ edges were the pre-L_III_-edges that were derived from the crystal field in response to the symmetry of the atoms surrounding Ca in the first shell. As shown in Figure 1, Kuzel’s salt, Friedel’s salt, and monosulfoaluminate had similar pre-L_III_-edge features with three distinguishable peaks because Ca atoms were seven-fold coordinated by the same type of ligands. In contrast, ettringite showed only two major pre-edge features because the Ca atoms were eight-fold coordinated. This observation was in good agreement with a previously reported study on the symmetry coordination and pre-edge features [39].

### 3.2. Phase Identification of NaCl-Reacted Monosulfoaluminate Sample Using XRD

**Figure 2:**
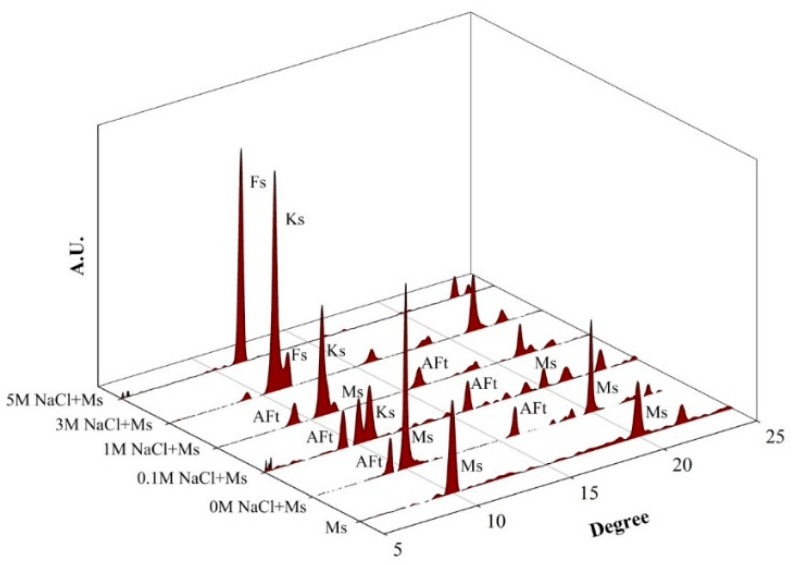
XRD patterns of the phase change measured from monosulfoaluminate samples suspended in NaCl solutions; Ks is Kuzel’s salt, Fs is Friedel’s salt, Ms is monosulfoaluminate, and AFt is ettringite.

Figure 2 presents the XRD results that show the phase changes of monosulfoaluminate from reacting with chloride ions in a series of samples, which were suspended in selected concentrations (0, 0.1, 1, 3, and 5 M) of NaCl solutions.

At the 0 M NaCl solution, although the diffraction pattern of the sample was dominated by the characteristic peaks of monosulfoaluminate, ettringite was also identified (see 0 M NaCl + Ms in Figure 2). The formation of ettringite was mainly due to a partial conversion of monosulfoaluminate to ettringite because monosulfoaluminate is metastable and easy to dissolve [24], even in an absence of chloride.

Even with a small concentration of 0.1 M NaCl solution, the peak intensities of monosulfoaluminate was significantly decreased with the appearance of the new peaks of Kuzel’s salt because the chloride concentration largely affects the phase change and the dissolution of monosulfoaluminate.

In the NaCl concentrations over 1 M, monosulfoaluminate was no longer detected. As the NaCl concentration increased, the peak intensities of ettringite were also reduced and almost disappeared at 5 M. The transformation of monosulfoaluminate to Friedel’s salt started from 3 M NaCl concentration and completed at 5 M.

### 3.3. Deconvolution of XANES and Thermodynamic Calculation

**Figure 3:**
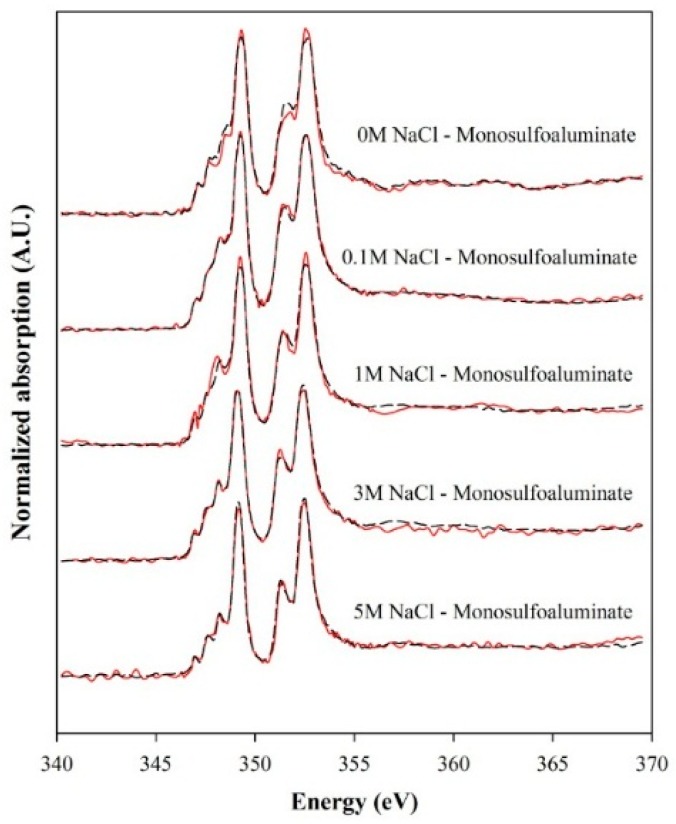
XANES spectra of the Ca L_III,II_-edge collected from monosulfoaluminate samples suspended in NaCl solutions. Solid lines are the measured XANES spectra. Broken lines are the LC fitted spectra.

Figure 3 shows the XANES spectra of monosulfoaluminate samples exposed to NaCl solutions for Ca L_III,II_-edge. Molar fractions of comprising phases in the NaCl-reacted samples were calculated using a least-square LC fitting method based on the Ca L_III,II_-edge reference spectra (Figure 1) of the identified phases in the XRD analysis. Figure 3 exhibits good fitted results. Table 2 summarizes the quantification results.

**Figure 4:**
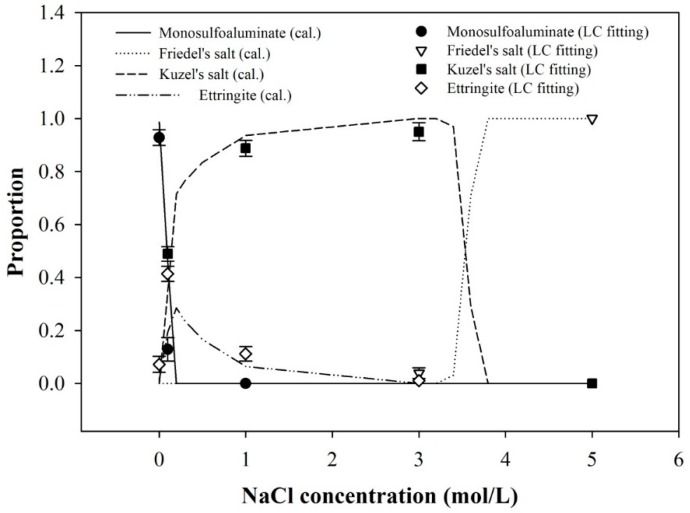
Calculated molar phase fractions from thermodynamic calculation (cal. in legend) and LC fitting of XANES spectra (LC fitting in legend) with different NaCl concentrations.

Figure 4 reports a sequence of the phase changes in the suspended samples in NaCl solutions with increasing concentrations from 0 to 5 M. In addition, the results of the thermodynamic calculation are given and compared with the quantification results from the deconvolution of the XANES spectra using the LC fitting. The thermodynamic calculation results showed a satisfactory agreement with those of the LC fitting for the XANES spectra.

### 3.4. STXM Results

**Figure 5:**
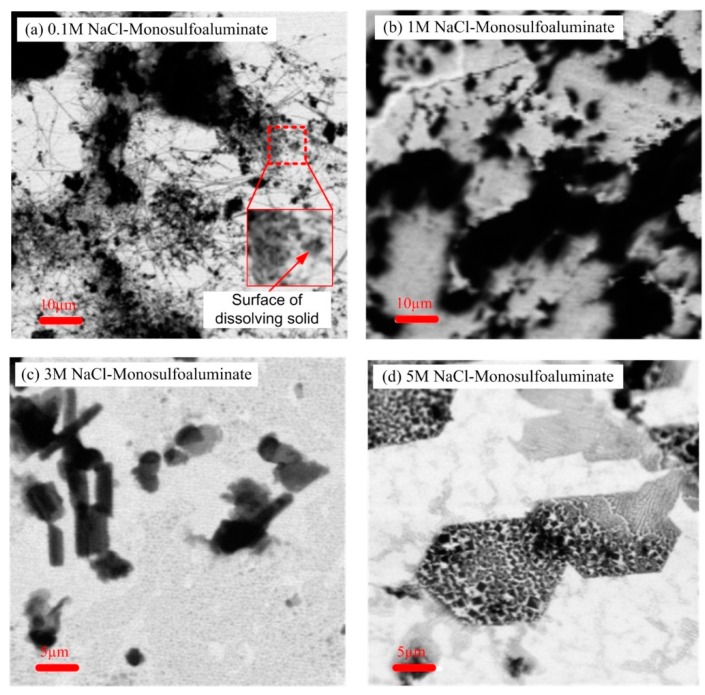
STXM images of NaCl-reacted monosulfoaluminate samples in: (**a**) 0.1 M; (**b**) 1 M; (**c**) 3 M; and (**d**) 5 M NaCl solutions.

Figure 5 presents the representative morphologies of the NaCl-reacted monosulfoaluminate samples observed in this study using STXM. The STXM images clearly showed morphological differences between low and high concentrations in NaCl solutions. At relatively low NaCl concentrations (*i.e.*, Figure 5a,b), the samples particles had blurry boundaries that were likely the dissolved surfaces of particles, while at relatively high NaCl concentrations (*i.e.*, Figure 5c,d), the samples only shows a very small portion of the dissolved boundaries; in particular, Figure 5d exhibited well-defined hexagonal plates with sharp edges, which were Friedel’s salt because its XRD pattern showed only the peaks of Friedel’s salt (see Figure 2). In addition, Figure 5a visibly displayed the presence of needle-like ettringite [2]. Although the XRD results indicated the samples up to 3 M NaCl concentration possessed ettringite, these needle-like compounds were not observable in Figure 5b,c.

## 4. Summary and Conclusions

Based on the aforementioned results, the process of phase change of monosulfoaluminate due to chloride binding is summarized as follows.

At low concentrations of NaCl solution (*i.e.*, ~0–0.1 M), a significant portion of monosulfoaluminate was dissolved into the NaCl aqueous solution. The STXM image in Figure 5a also revealed the dissolution of monosulfoaluminate, followed by the ettringite formation. As the dissolution of monosulfoaluminate released a lot of sulfate ions, the sulfate concentration was likely to be elevated in the solution. As ettringite was a more stable phase than monosulfoaluminate in the sulfate-rich environment, ettringite formed at the low NaCl concentrations. Likewise, the thermodynamic calculations and the LC fitting results also demonstrated the fractional increase of ettringite up to 0.1 M NaCl (see Figure 4). At 0.1 M NaCl solution, apart from the dissolution of monosulfoaluminate, the quantity of monosulfoaluminate was also simultaneously reduced through the direct transformation of monosulfoaluminate to Kuzel’s salt by the ion exchange of sulfate ions in interlayer spaces with free chloride ions in the NaCl aqueous solution.

At intermediate concentrations of NaCl solution (*i.e.*, ~1–3 M), the fraction of Kuzel’s salt gradually increased with the substantial decrease of ettringite, and monosulfoaluminate was no longer present (see Figure 4). In this concentration range, it seems that the dissolution of monosulfoaluminate became suppressed as the NaCl concentration increased given that: (1) the sample in 3 M NaCl solution (Figure 5c) showed greatly reduced portion of dissolved blurry boundaries of particles compared to that in 1 M NaCl solution (Figure 5b); and (2) the reduced ettringite formation generally occurs when dissolution/precipitation process is prohibited [40]. Therefore, monosulfoaluminate mostly disappeared because of its direct phase transition to Kuzel’s salt mainly due to the ion exchange rather than the dissolution process at this concentration range.

At the high concentration of NaCl solution (*i.e.*, ~5 M), the further increase of NaCl concentration produced only Friedel’s salt. Note that Figure 5d barely presented the indication of particle dissolution and ettringite formation, but only well-defined hexagonal plate of Friedel’s salts. Thus, the ion exchange was likely to be the only binding mechanism of chloride ions, and it was responsible for the phase transition from monosulfoaluminate to Friedel’s salt at this concentration.

Phase modification of monosulfoaluminate due to the ion exchange in a solution may depend on pressure, temperature, mass balances and chemical activity coefficients. Thus, the results obtained herein might only be valid in a specific range of aqueous NaCl concentration. Nevertheless, understanding of the phase change of monosulfoaluminate as a function of NaCl concentration is important to develop reliable adsorption isotherms because it is needed to properly predict when steel corrosion of reinforced concretes will initiate in marine environments.

Lastly, in this study, the thermodynamic calculation was conducted under the assumption that no solid solution existed between monosulfoaluminate and Friedel’s salt. Thus, the good agreement between the XANES results and the thermodynamic calculation in Section 3.3 supports the conclusion of Balnois *et al.* [13] rather than that of Mesbah *et al.* [23] on the existence of the solid solution.

## Figures and Tables

**Table 1:** Ca L_III,II_-edge XANES intensity ratios and peak positions of Friedel’s salt, Kuzel’s salt, monosulfoaluminate, and ettringite, and coordination within the first shell of calcium (N.D.: Not Detected).

Mineral Name		AFm Phase			AFt Phase
		Friedel’s Salt	Kuzel’s Salt	MonosulfoalumInate	Ettringite
Intensity ratio	*a*_2_/*a*_1_	2.77	1.36	1.87	2.56
	*b*_2_/*b*_1_	1.86	1.23	1.93	1.70
Peak position (eV)	Pre-L_III_-edge	346.9 347.6 348.2	347.0 347.6 348.3	347.1 347.8 348.6	347.2 348.3
	L_III_-edge	349.2	349.2	349.3	349.4
	Pre-L_II_-edge	351.3	351.37	351.6	351.6
	L_II_-edges	352.5	352.57	352.6	352.6
	Post-edge	357.3	N.D.	359.0362.1	N.D.
Atomic distribution in the first shell of Ca		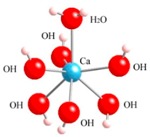			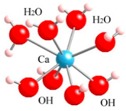

N.D.: Not Detected.

**Table 2:** Molar phase fractions determined from the LC fitting of Ca L_III,II_-edge XANES spectra.

NaCl Solution	Monosulfoaluminate	Friedel’s Salt	Kuzel’s Salt	Ettringite	*R*-Factor
0.0 M	0.928 ± 0.030	N.D.	N.D.	0.072 ± 0.030	0.0061
0.1 M	0.129 ± 0.044	N.D.	0.489 ± 0.027	0.414 ± 0.028	0.0011
1.0 M	0 ± 0	N.D.	0.888 ± 0.030	0.112 ± 0.027	0.0055
3.0 M	N.D.	0.039 ± 0.020	0.950 ± 0.034	0.011 ± 0.010	0.0022
5.0 M	N.D.	1 ± 0	0 ± 0	N.D.	0.0042

Note: *R*-factor smaller than 0.02 indicates a good degree of fitness; N.D.: Not Detected.

## References

[1] Gjørv O.E. (2002). Durability and Service Life of Concrete Structures.

[2] Mehta P.K., Monteiro P.J.M. (2006). Concrete: Microstructure, Properties, and Materials.

[3] Brown P., Bothe J. (2004). The system CaO-Al_2_O_3_-CaCl_2_-H_2_O at 23 ± 2 °C and the mechanisms of chloride binding in concrete. Cem. Concr. Res..

[4] Csizmadia J., Balözs G., Tamás F.D. (2001). Chloride ion binding capacity of aluminoferrites. Cem. Concr. Res..

[5] Delagrave A., Marchand J., Ollivier J.P., Julien S., Hazrati K. (1997). Chloride binding capacity of various hydrated cement paste systems. Adv. Cem. Based Mater..

[6] Hirao H., Yamada K., Takahashi H., Zibara H. (2005). Chloride binding of cement estimated by binding isotherms of hydrates. J. Adv. Concr. Technol..

[7] Luping T., Nilsson L.O. (1993). Chloride binding capacity and binding isotherms of OPC pastes and mortars. Cem. Concr. Res..

[8] Suryavanshi A.K., Scantlebury J.D., Lyon S.B. (1995). The binding of chloride ions by sulphate resistant Portland cement. Cem. Concr. Res..

[9] Tritthart J. (1989). Chloride binding in cement II. The influence of the hydroxide concentration in the pore solution of hardened cement paste on chloride binding. Cem. Concr. Res..

[10] Xu Y. (1997). The influence of sulphates on chloride binding and pore solution chemistry. Cem. Concr. Res..

[11] Yuan Q., Shi C., De Schutter G., Audenaert K., Deng D. (2009). Chloride binding of cement-based materials subjected to external chloride environment-a review. Constr. Build. Mater..

[12] Zibara H., Hooton D., Yamada K., Thomas M.D.A. (2002). Roles of cement mineral phases in chloride binding. Cem. Sci. Conc. Technol..

[13] Balonis M., Lothenbach B., Le Saout G., Glasser F.P. (2010). Impact of chloride on the mineralogy of hydrated Portland cement systems. Cem. Concr. Res..

[14] Glasser F.P., Kindness A., Stronach S.A. (1999). Stability and solubility relationships in AFm phases: Part I. chloride, sulfate and hydroxide. Cem. Concr. Res..

[15] Suryavanshi A.K., Scantlebury J.D., Lyon S.B. (1996). Mechanism of Friedel’s salt formation in cements rich in tri-calcium aluminate. Cem. Concr. Res..

[16] Jones M.R., Macphee D.E., Chudek J.A., Hunter G., Lannegrand R., Talero R., Scrimgeour S.N. (2003). Studies using ^27^Al MAS NMR of AFm and AFt phases and the formation of Friedel’s salt. Cem. Concr. Res..

[17] Birnin-Yauri U.A., Glasser F.P. (1998). Friedel’s salt, Ca_2_Al(OH)_6_(Cl,OH)·2H_2_O: Its solid solutions and their role in chloride binding. Cem. Concr. Res..

[18] Mesbah A., Cau-dit-Coumes C., Renaudin G., Frizon F., Leroux F. (2012). Uptake of chloride and carbonate ions by calcium monosulfoaluminate hydrate. Cem. Concr. Res..

[19] Ipavec A., Vuk T., Gabrovšek R., Kaučič V. (2013). Chloride binding into hydrated blended cements: The influence of limestone and alkalinity. Cem. Concr. Res..

[20] Zhang M., Chen J., Lv Y., Wang D., Ye J. (2013). Study on the expansion of concrete under attack of sulfate and sulfate–chloride ions. Constr. Build. Mater..

[21] De Weerdt K., Colombo A., Coppola L., Justnes H., Geiker M. (2015). Impact of the associated cation on chloride binding of Portland cement paste. Cem. Concr. Res..

[22] Galan I., Perron L., Glasser F.P. (2015). Impact of chloride-rich environments on cement paste mineralogy. Cem. Concr. Res..

[23] Matschei T., Lothenbach B., Glasser F. (2007). The AFm phase in Portland cement. Cem. Concr. Res..

[24] Perkins R.B., Palmer C.D. (1999). Solubility of ettringite (Ca_6_[Al(OH)_6_]_2_(SO_4_)_3_·26H_2_O) at 5–75 °C. Geochim. Cosmochim. Acta.

[25] (2006). X’Pert HighScore.

[26] (2006). AXis 2000-Analysis of X-ray Images and Spectra.

[27] Ha J., Chae S., Chou K.W., Tyliszczak T., Monteiro P.J.M. (2012). Effect of polymers on the nanostructure and on the carbonation of calcium silicate hydrates: A scanning transmission X-ray microscopy study. J. Mater. Sci..

[28] Ravel B., Newville M. (2005). ATHENA, ARTEMIS, HEPHAESTUS: Data analysis for X-ray absorption spectroscopy using IFEFFIT. J. Synchrotron Radiat..

[29] (1999). A Computer Program for Speciation, Batch-Reaction, One-Dimensional Transport, and Inverse Geochemical Calculation.

[30] Bond K.A., Heath T.G., Tweed C.J. (1997). HATCHES: A referenced thermodynamic database for chemical equilibrium studies. Nirex Rep..

[31] Chatterji S. (1978). Mechanism of the CaCl_2_ attack on Portland cement concrete. Cem. Concr. Res..

[32] Renaudin G., Kubel F., Rivera J.P., Francois M. (1999). Structural phase transition and high temperature phase structure of Friedels salt, 3CaO∙Al_2_O_3_∙CaCl_2_∙10H_2_O. Cem. Concr. Res..

[33] Rapin J.P., Renaudin G., Elkaim E., Francois M. (2002). Structural transition of Friedel’s salt 3CaO∙Al_2_O_3_∙CaCl_2_∙10H_2_O studied by synchrotron powder diffraction. Cem. Concr. Res..

[34] Andersen M.D., Jakobsen H.J., Skibsted J. (2002). Characterization of the α-β phase transition in Friedel’s salt (Ca_2_Al(OH)_6_Cl∙2H_2_O) by variable-temperature ^27^Al MAS NMR spectroscopy. J. Phys. Chem. A.

[35] Balonis M., Glasser F.P. (2009). The density of cement phases. Cem. Concr. Res..

[36] Bothe J.V. (2004). PHREEQC modeling of Friedel’s salt equilibria at 23 ± 1 °C. Cem. Concr. Res..

[37] Wieland E., Dähn R., Vespa M., Lothenbach B. (2010). Micro-spectroscopic investigation of Al and S speciation in hardened cement paste. Cem. Concr. Res..

[38] Naftel S.J., Sham T.K., Yiu Y.M., Yates B.W. (2001). Calcium L-edge XANES study of some calcium compounds. J. Synchrotron Radiat..

[39] Newville M. (2001). IFEFFIT: Interactive XAFS analysis and FEFF fitting. J. Synchrotron Radiat..

[40] Taylor H.F.W., Famy C., Scrivener K.L. (2001). Delayed ettringite formation. Cem. Concr. Res..

